# Shear Stress Counteracts Endothelial CX3CL1 Induction and Monocytic Cell Adhesion

**DOI:** 10.1155/2017/1515389

**Published:** 2017-03-26

**Authors:** Aaron Babendreyer, Lisa Molls, Daniela Dreymueller, Stefan Uhlig, Andreas Ludwig

**Affiliations:** Institute of Pharmacology and Toxicology, RWTH Aachen University, Aachen, Germany

## Abstract

Flow conditions critically regulate endothelial cell functions in the vasculature. Reduced shear stress resulting from disturbed blood flow can drive the development of vascular inflammatory lesions. On endothelial cells, the transmembrane chemokine CX3CL1/fractalkine promotes vascular inflammation by functioning as a surface-expressed adhesion molecule and by becoming released as soluble chemoattractant for monocytic cells expressing the receptor CX3CR1. Here, we report that endothelial cells from human artery, vein, or microvasculature constitutively express CX3CL1 when cultured under static conditions. Stimulation with TNF*α* under static or very low shear stress conditions strongly upregulates CX3CL1 expression. By contrast, CX3CL1 induction is profoundly reduced when cells are exposed to higher shear stress. When endothelial cells were grown and subsequently stimulated with TNF*α* under low shear stress, strong adhesion of monocytic THP-1 cells to endothelial cells was observed. This adhesion was in part mediated by transmembrane CX3CL1 as demonstrated with a neutralizing antibody. By contrast, no CX3CL1-dependent adhesion to stimulated endothelium was observed at high shear stress. Thus, during early stages of vascular inflammation, low shear stress typically seen at atherosclerosis-prone regions promotes the induction of endothelial CX3CL1 and monocytic cell recruitment, whereas physiological shear stress counteracts this inflammatory activation of endothelial cells.

## 1. Introduction

In the body, vascular endothelial cells are constantly exposed to blood flow. The resulting laminar shear stress on the endothelial surface can largely differ in large and small arteries or veins, respectively [1, 2]. Exposure to laminar flow typically leads to an alignment of the endothelial cells in the direction of the flow, cytoskeletal rearrangements, the formation of tight endothelial cell-to-cell contacts, reduced permeability, arrest of cell proliferation, and prolonged cell survival [3–7]. These phenotypical characteristics are maintained by a transcriptional response to the flow conditions resulting in the altered expression of regulators of the vascular tone, endothelial surface molecules, and soluble mediators. Typically, endothelial NO synthase is upregulated by high shear stress, whereas endothelin-1 or vascular cell adhesion molecule-1 (VCAM-1) gene expression is downregulated [8–12].

The importance of shear stress for endothelial cell functions is highlighted by pathological processes associated with reduced or absent laminar shear stress, which can occur in vascular beds that are prone to atherosclerosis. At branch points and curved areas such as the carotid sinus, disturbed flow including flow reversal or turbulence may occur. In these areas, the average shear stress is considerably lower and values below 1 dyn/cm^2^ can occur, which is 10 times lower than the average of 10 dyn/cm^2^ in the human vasculature and even 100 times lower compared to the microvasculature [1, 2, 13]. These areas are characterized by an absence of preferential endothelial alignment and are predisposed to develop atherosclerotic lesions. An early step in the development of atherosclerotic lesions is the production of proinflammatory chemokines and adhesion molecules by the vascular wall. This results in increased binding of inflammatory leukocytes, especially monocytes, from the blood to the vascular surface. The adherent monocytes become activated and migrate into the vascular wall where they contribute to the lesion development by production of inflammatory mediators, by uptake of lipids, and by differentiation into macrophages and finally foam cells [14].

Monocyte recruitment to atherosclerotic lesions is driven by several chemotactic cytokines including CX3CL1 also termed fractalkine [15]. CX3CL1 is special within the chemokine family, since it is synthesized as a transmembrane molecule that is expressed on the endothelial cell surface [16]. As such, the chemokine can interact with its receptor CX3CR1, which is expressed on monocytic cells, T cell subsets, and NK cells [17]. Thereby, CX3CL1 can promote cell adhesion without the need of further activation [18]. Stimulation of cultured endothelial cells with proinflammatory mediators leads to enhanced expression of CX3CL1, which then promotes adhesion of monocytic cells. Thereafter, the activation of CX3CR1 can induce cell migration of the adherent monocytes leading to transmigration through the endothelial cell layer [19]. In addition to its activity as transmembrane adhesion molecule, CX3CL1 can act as classical chemoattractant for leukocytes when it is shed from the cell surface by the activity of ADAM10 (short for a disintegrin and metalloproteinase) and ADAM17 [20, 21]. Besides CX3CL1, the only other transmembrane chemokine is CXCL16, which is also expressed and shed by endothelial cells [22, 23] and implicated in the recruitment of T cells to the inflamed vasculature [24, 25].

It has been shown that CX3CL1 is highly expressed at vascular sites, which are prone to lesion development and serve as one of the critical mediators driving monocytic cell recruitment to these lesions [26, 27]. In carotid arteries, CX3CL1 expression is highest at regions of low laminar shear stress [28]. This may suggest that the upregulation of the chemokine can be a direct result of the altered flow conditions in these regions. In the present report, we further address this question by performing flow experiments with cultured endothelial cells. We show that endothelial cells of different vascular beds respond to flow conditions by downregulating the gene expression of CX3CL1. Moreover, flow conditions suppress the induction of CX3CL1 in response to TNF*α*. We then show that the reduced surface expression of CX3CL1 on endothelial cells results in reduced adhesion of monocytic cells. Thus, physiological shear stress acts as atheroprotection by downregulating CX3CL1 expression to maintain a noninflammatory phenotype of endothelial cells.

## 2. Material and Methods

### 2.1. Antibodies, Chemokines, and Inhibitors

Biotinylated mouse monoclonal antibody against human CX3CL1 (clone 51637), mouse monoclonal antibody against human CX3CL1 (clone 51637 and clone 81506), goat polyclonal antibody against human CXCL16, and mouse IgG1 isotype control were from R&D Systems (Wiesbaden, Germany). Biotinylated rabbit polyclonal antibody against human CXCL16, human CX3CL1, human CXCL16, and human TNF*α* were from PeproTech (Rocky Hill, USA). Allophycocyanin- (APC-) conjugated goat antimouse antibody was from Jackson ImmunoResearch Laboratories, Inc. (West Grove, USA). Calcein-AM was from BIOTREND Chemikalien GmbH (Cologne, Germany).

### 2.2. Cell Culture

Monocytic THP-1 cells were cultured in RPMI (Sigma-Aldrich, St. Louis, USA) with 10% FBS (PAN-Biotech, Aidenbach, Germany) with a cell density of 0.2 to 2.0 × 10^6^ cells per ml.

Human pulmonary microvascular endothelial cells (HPMECs) were from PromoCell (Heidelberg, Germany) and cultured in Endothelial Cell Growth Medium MV2 (PromoCell) as recommended by the supplier. Human umbilical vein endothelial cells (HUVECs) and human umbilical artery endothelial cells (HUAECs) were isolated from the umbilical cord of caesarean sections in our laboratory as described [19] and cultured in Endopan-3 from PAN-Biotech (Aidenbach, Germany).

For flow experiments, cells were used in passages 4 to 6 and seeded with a density of 100,000 cells/cm^2^ in ibidi *μ*-Slides of different types (0.1, 0.2, 0.4, and 0.8 mm *μ*-Slides, Martinsried, Germany) or 24 well plates for the static control. After 24 h, supernatants and cells were harvested or stimulated with 10 ng/ml TNF*α* for another 24 h. The flow in the *μ*-Slides was created with the ibidi pump system. Depending on the geometry of the *μ*-Slide type, the flow rate was adjusted as specified by the manufacturer to yield the desired laminar shear stress.

### 2.3. RT-qPCR

The mRNA levels for CX3CL1 and CXCL16 were quantified by RT-qPCR analysis and normalized to the mRNA level of GAPDH (glyceraldehyde 3-phosphate dehydrogenase). For the first set of flow experiments, a set of housekeeping genes (GAPDH, TATA-binding protein—TBP, and cytochrome C1—CYC1) were used as reference genes instead of only GAPDH. RNA was extracted using RNeasy Kit (Qiagen, Hilden, Germany) and quantified using NanoDrop (Peqlab, Erlangen, Germany). Equal amounts of mRNA within each data set were reversely transcribed using PrimeScript™ RT Reagent Kit (Takara Bio Europe, St-Germain-en-Laye, France) according to the manufacturer's protocol. PCR reactions were performed using SYBR Premix Ex Taq II (Takara Bio Europe) according to the manufacturer's protocol. The following primers were used with the specific primer annealing time given in brackets: *CX3CL1* (forward: GGTTAGGCATTGTGGGAAGG, reverse: AGATGGGAGTATGTTGGTGG) (60°C), *CXCL16* (forward: TGTCTATACTACACGAGGTTCCA, reverse: AGCATGTCCACATTCTTTGAG) (60°C), *GAPDH* (forward: CGGGGCTCTCCAGAACATCATCC, reverse: CCAGCCCCAGCGTCAAAGGTG) (66°C), *TBP* (forward: GAGCCAAGAGTGAAGAACAGTC, reverse: GCTCCCCACCATATTCTGAATCT) (60°C), and *CYC1* (forward: AGCTATCCGTGGTCTCACC, reverse: CCGCATGAACATCTCCCCATC) (59°C). All PCR reactions were run on a LightCycler® 480 System (Roche, Basel, Switzerland) with the following protocol: 45 cycles of 10 s denaturation at 95°C, followed by 10 s annealing at the indicated temperature, and 15 s amplification at 72°C. Standard curves were determined by a serial dilution of a defined cDNA standard within each data set. Relative quantification was performed with the E-Method from Roche Applied Bioscience using the LightCycler®480 software 1.5 (Roche).

### 2.4. Flow Cytometric Analysis

PBS supplemented with 0.2% BSA was used as assay buffer, and all steps of the staining process were performed at 4°C. HUVECs were analyzed for expression of CX3CL1 by incubation with mouse monoclonal antibodies against human CX3CL1 (5 *μ*g/ml) followed by incubation with APC-conjugated antimouse antibody (1 : 200). Isotype controls were used in parallel. The fluorescence signal was detected by flow cytometry (LRS Fortessa, BD Biosciences) and analyzed with FlowJo 8.7.3 software (Tree Star, Inc., Ashland, USA).

### 2.5. Apoptosis Assay

Endothelial cells were incubated with FITC-labelled annexin V in Hank's buffered salt solution (HBSS) for 30 min. After washing with HBSS, at least 8 images of each well or *μ*-Slide were made with the automated IncuCyte ZOOM microscope (Essen BioScience, Ann Arbor, USA). The relative apoptosis was determined by the ratio of the green fluorescence and the total cell confluence. The calculation of the green confluence and the total cell confluence was performed by the *IncuCyte ZOOM microscope* software 2014A (Essen BioScience).

### 2.6. ELISA Measurements

The release of human CX3CL1 and CXCL16 into the supernatant was analyzed by ELISA. Before the measurement, the culture supernatants were concentrated from 3 ml to 0.5 ml using Vivaspin 6 columns (10.000 MWCO) (Sartorius, Göttingen, Germany). 96 well plates were coated in PBS overnight at 4°C with a capture antibody against CX3CL1 (4 *μ*g/ml) or CXCL16 (1 *μ*g/ml), respectively. Before adding the samples, unspecific binding to the plate was blocked with washing buffer (PBS + 0.05% Tween) containing 2% BSA for 2 h at room temperature. Samples were incubated for another 2 h at room temperature, and the bound chemokines were detected with a biotinylated secondary antibody against CX3CL1 (0.3 *μ*g/ml) or CXCL16 (0.5 *μ*g/ml). The chromogenic reaction was mediated by a standard procedure using 0.1 U/ml streptavidin-conjugated horseradish peroxidase (Roche, Basel, Switzerland) and the BM Blue POD substrate (Roche).

### 2.7. Flow Adhesion Assay

HUVECs were cultured for 24 h under flow conditions (0.5 or 30 dyn/cm^2^) and stimulated with 10 ng/ml TNF*α* or left untreated for another 24 h. In some experiments, cultivation was continued in the presence of an antibody against human CX3CL1 or an IgG control antibody (10 *μ*g/ml) for 30 minutes at 37°C. Subsequently, HUVECs were rinsed with flow adhesion buffer (10% HBSS, 1% HEPES, and 1% BSA in H_2_O) for 3 minutes with a shear stress of 6 dyn/cm^2^. THP-1 cells were labelled for 30 min with 0.5 *μ*M Calcein AM, washed with PBS, and resuspended at a concentration of 0.5 × 10^6^ cells in flow adhesion buffer. Labelled THP-1 cells were then perfused over the HUVECs with a flow rate of 1 ml/min. Images of the *μ*-Slides were taken during the perfusion, and adherent THP-1 cells were quantified using the IncuCyte ZOOM (Essen BioScience, Ann Arbor, USA). For each experiment, 8 images were taken and the average count per picture was used as result.

### 2.8. Statistics

Quantitative data are shown as mean + SD calculated from at least three independent experiments and for HUVECs and HUAECs also from different isolations. Data were analyzed by general mixed model analysis (PROC GLIMMIX, SAS 9.4, SAS Institute Inc., Cary, USA) and assumed to be derived from either log normal, negative binomial (count data), or beta (percentage data) distributions; residual plots and the Shapiro-Wilk test were used as diagnostics. In case of heteroscedasticity (according to the covtest statement), the degrees of freedom were adjusted by the Kenward-Roger approximation. The false discovery rate (FDR) was used to correct for multiple comparisons. Diagrams were created with PRISM 5.0 (GraphPad Software, La Jolla, USA). A *p* value < 0.05 was always considered significant.

## 3. Results

### 3.1. Endothelial CX3CL1 mRNA and Protein Expression Is Downregulated under Flow Conditions

**Figure 1:**
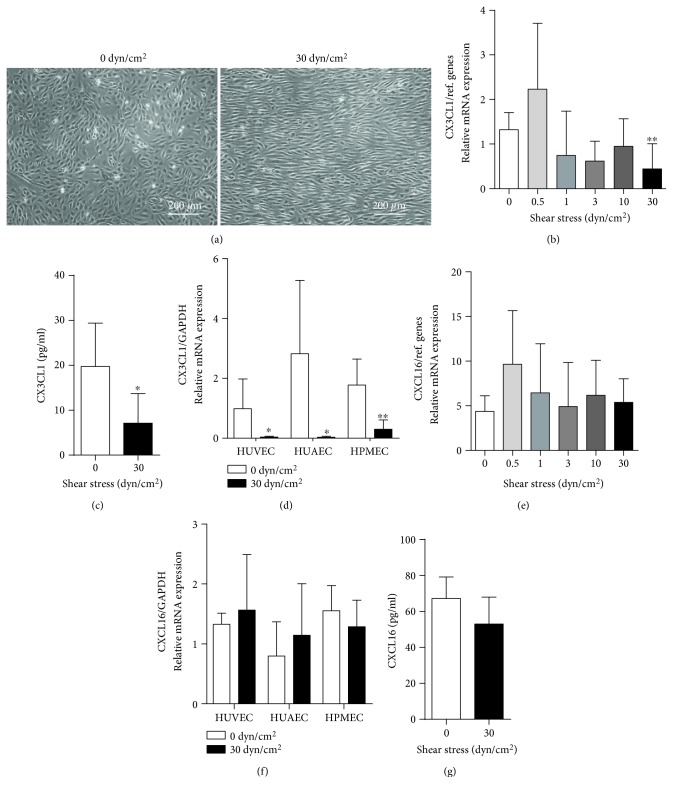
Regulation of constitutive CX3CL1 expression by shear stress. (a–c) HUVECs were cultured for 24 h under different conditions of flow resulting in the indicated laminar shear stress. Cells were then analyzed for their morphology by microscopy (a), for CX3CL1 mRNA expression (b), and their release of soluble CX3CL1 into the supernatant (c). (e, g) HUVECs were cultured under different flow conditions as described for (a–c) and analyzed for CXCL16 mRNA expression (e) and their release of soluble CXCL16 into the supernatant (g). (d, f) Endothelial cells from different vascular beds were cultured for 24 h with and without flow and analyzed for their CX3CL1 (d) or CXCL16 (f) mRNA expression. Data in (b–g) are shown as mean + SD for at least five different experiments. Statistical differences in comparison to the static control are indicated by asterisks (^∗^*p* ≤ 0.05, ^∗∗^*p* ≤ 0.01).

Human umbilical vein endothelial cells (HUVECs) were cultured under static or flow conditions. The resulting laminar shear stress at the endothelial surface was calculated from the different flow rates and flow chamber geometries, according to the specifications of the manufacturer. As expected, endothelial cells under flow conditions acquired an elongated shape and aligned in the direction of flow (Figure 1(a)). Analysis of CX3CL1 mRNA expression revealed that the chemokine is expressed to some degree under static culture conditions or conditions with low shear stress (0.5 dyn/cm^2^). This expression is downregulated when the cells were exposed to higher shear stress (Figure 1(b)). Consistent with this transcriptional regulation, released CX3CL1 protein was detected in the supernatant of static HUVEC culture, but considerably less CX3CL1 was found in supernatants from cultures exposed to higher shear stress (Figure 1(c)). These observations were made with HUVECs from at least five different donors.

To investigate whether the observed regulation of CX3CL1 would depend on the type and source of endothelial cells, we compared HUVECs with human umbilical artery endothelial cells (HUAECs) and human pulmonary microvascular endothelial cells (HPMECs). For all cell types, we observed constitutive expression of CX3CL1 mRNA when cells were cultured under static conditions. This expression was considerably decreased, when cells were exposed to high shear stress (Figure 1(d)).

We also studied the regulation of the other structurally related transmembrane chemokine CXCL16 in endothelial cells. This chemokine is also expressed at a constitutive level by endothelial cells, but the mRNA and protein expression is not affected by flow conditions. Further, there is no difference in HUVEC, HUAEC, or HPMECs (Figures 1(e)–1(g)).

These findings show that flow conditions downregulate the basal expression of CX3CL1 but not that of CXCL16 in endothelial cells from different vascular beds.

### 3.2. Inflammatory Induction of Endothelial CX3CL1 Is Suppressed under Flow Conditions

**Figure 2:**
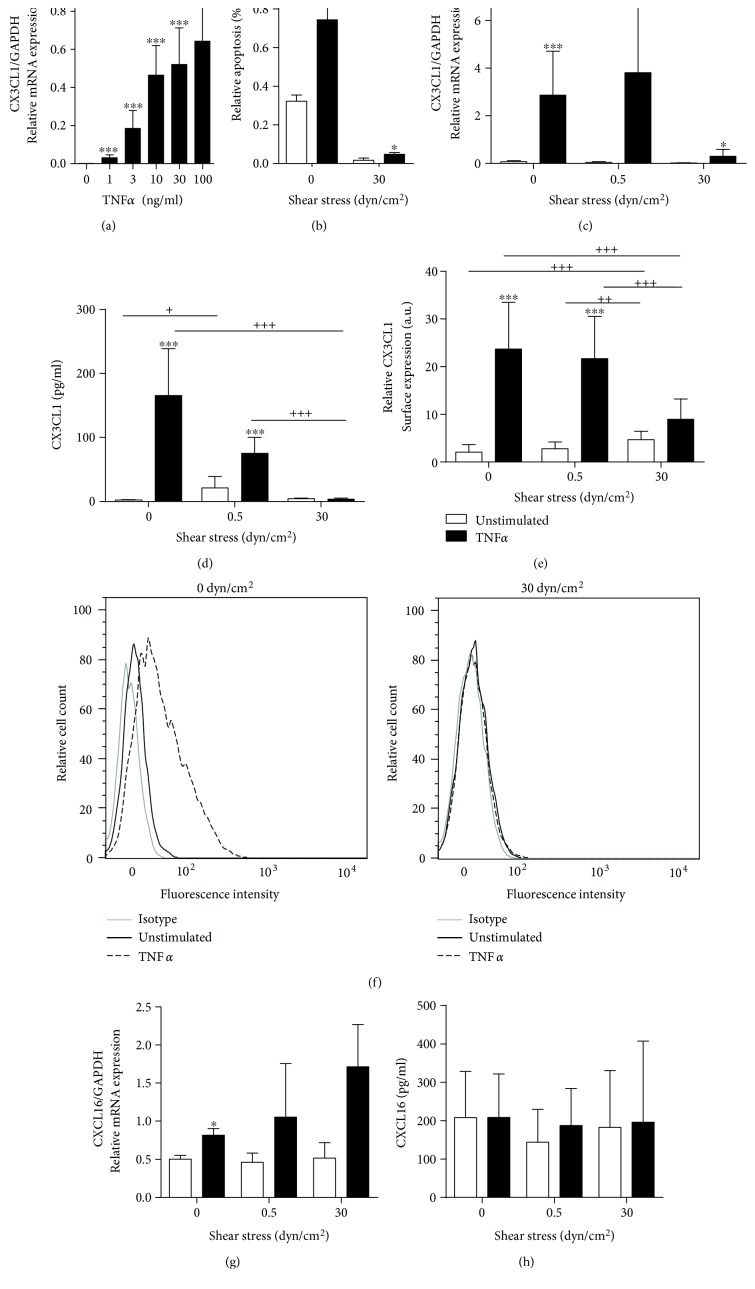
Regulation of induced CX3CL1 expression by shear stress. (a) HUVECs were stimulated with the indicated concentrations of TNF*α* for 24 h and analyzed for CX3CL1 mRNA expression. (b–f) HUVECs were cultured for 24 h and subsequently stimulated with or without TNF*α* for 24 h at the indicated levels of shear stress. TNF*α*-induced apoptosis was detected by annexin V staining (b). Cells were also analyzed for CX3CL1 mRNA expression (c) and release of CX3CL1 into the supernatant (d). CX3CL1 surface expression levels were determined by flow cytometry and are shown as summary of median fluorescence intensities and as representative histograms (e, f). (g, h) HUVECs were cultured and stimulated as described and analyzed for CXCL16 mRNA expression (g) and release of CXCL16 into the supernatant (h). Data are shown as mean + SD and are representative for at least three (b, d, h) or four (a, c, e, g) different experiments. Statistical differences to the unstimulated control are indicated by asterisks (^∗^*p* ≤ 0.05, ^∗∗^*p* ≤ 0.01, and ^∗∗∗^*p* ≤ 0.001) and differences between the flow conditions are indicated by crosses (^+^*p* ≤ 0.05, ^++^*p* ≤ 0.01, and ^+++^*p* ≤ 0.001).

Stimulation with proinflammatory cytokines such as TNF*α* has been reported to strongly upregulate the expression of CX3CL1 in endothelial cells [16]. We therefore questioned how flow conditions would interfere with the upregulation of CX3CL1 expression in response to this cytokine. TNF*α* dose dependently induced CX3CL1 mRNA in endothelial cells (Figure 2(a)). A suboptimal dosage of 10 ng/ml TNF*α* was then chosen for flow experiments. As detected by annexin V staining, TNF*α*-induced endothelial apoptosis was minimal under static conditions (less than 0.5%) and almost absent under flow (Figure 2(b)).

Compared to static conditions, the upregulation of CX3CL1 by TNF*α* was clearly suppressed at higher shear stress but not at low shear stress (Figure 2(c)). This suppression of mRNA expression correlated with a profound reduction of soluble CXC3L1 release into the supernatant and with a diminished surface expression of the transmembrane chemokine at conditions of higher shear stress (Figures 2(d) and 2(e)).

Notably, CXCL16 mRNA expression is only slightly upregulated by TNF*α* under static culture conditions. In contrast to CX3CL1, which is downregulated by flow conditions, CXCL16 seems to be slightly upregulated under flow conditions when cells are stimulated with TNF*α* (Figure 2(g)). However, this upregulation was not seen on the level of CXCL16 protein release (Figure 2(h)), which may be due to the high variability of CXCL16 release from the HUVEC preparations of the different donors and the fact that there is already a strong constitutive release of CXCL16, which might override the slight induction.

The data indicate that the exposure of endothelial cells to high shear stress can suppress the acquisition of a proinflammatory phenotype with respect to the induction of CX3CL1, but this does not account for CXCL16.

### 3.3. Monocytic Cell Adhesion to Activated Endothelial Cells Is Abrogated under Flow Conditions

Transmembrane CX3CL1 expressed on the endothelial surface can bind to its receptor CX3CR1 on monocytic cells and thereby mediate tight flow-resistant adhesion between both cell types. Cultured THP-1 cells express CX3CR1 and can be used as model for monocytic cell adhesion to endothelial cell-expressed CX3CL1 [19, 29]. We sought to investigate how flow conditions would affect THP-1 cell adhesion and whether this is affected by the regulation of CX3CL1. HUVECs were grown and stimulated with or without TNF at flow conditions with low and high shear stress. Subsequently, THP-1 cells were perfused over the endothelial cells with the same flow rate for all conditions.

**Figure 3:**
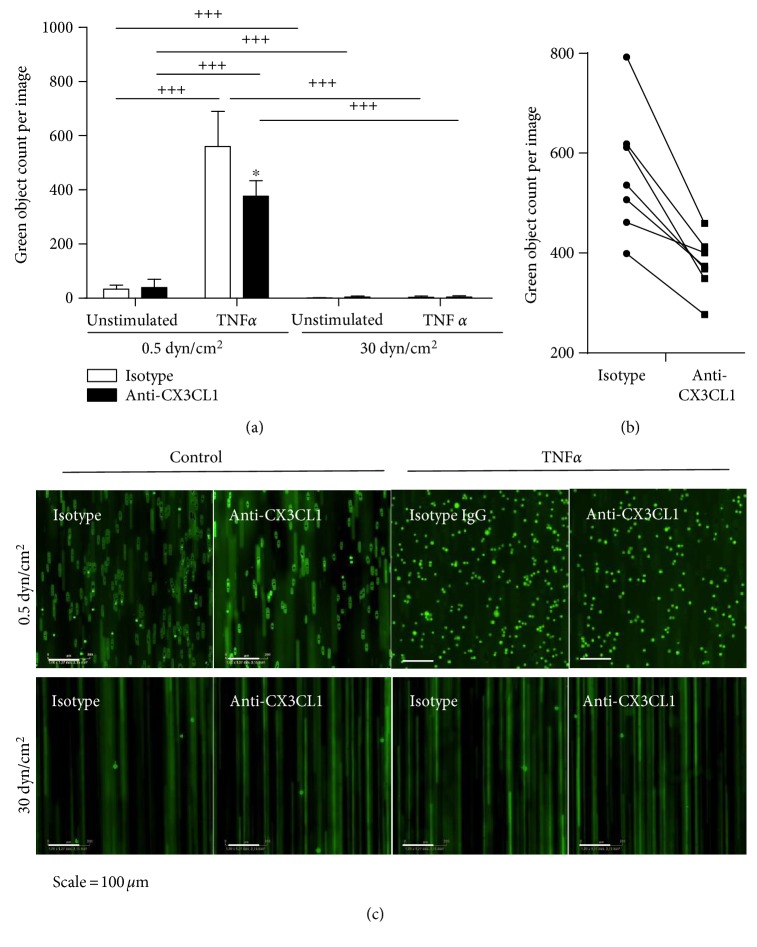
Regulation of CX3CL1 dependent THP-1 cell adhesion by shear stress. (a–c) HUVECs were cultured for 24 h and subsequently stimulated with or without TNF*α* for 24 h at the indicated levels of shear stress. Cells were then treated with a neutralizing antibody against CX3CL1 or an IgG isotype control for 0.5 h. Subsequently, fluorescently labelled THP-1 cells were perfused over the endothelial cell layer. THP-1 cells were visualized by fluorescence microscopy, and for each experiment adhered, THP-1 cells were counted in at least 8 images (a). Data are shown as mean + SD of 3–8 experiments as indicated. Statistical differences in comparison to the IgG isotype control or the specified flow conditions are indicated by asterisks (^∗^*p* ≤ 0.05) or crosses (^+++^*p* ≤ 0.001), respectively. (b) The effect of the inhibitory antibody against CX3CL1 is shown for each experiment performed with a different endothelial isolate. (c) Representative images showing adherent THP-1 cells (green bright circular objects) and flowing THP-1 cells (green lines) for all conditions.

Only low adhesion was observed when endothelial cells were cultured at low shear stress and left unstimulated, but stimulation with TNF*α* profoundly enhanced THP-1 cell adhesion (Figures 3(a) and 3(c)). This adhesion was at least in part mediated by CX3CL1 as evidenced by the incubation of the endothelial cells with a neutralizing antibody to CX3CL1 prior to adhesion experiments. This antibody-mediated inhibition reduced about one third of cell adhesion and was consistently observed in all experiments (Figure 3(b)).

When endothelial cells were grown and stimulated at conditions of high shear stress, THP-1 cell adhesion was almost completely abolished and no inhibitory effect of the neutralizing antibody could be observed (Figures 3(a) and 3(c)). These results demonstrate that culture under conditions of low pathologically relevant shear stress results in profound induction of cell adhesion upon proinflammatory stimulation. This adhesion is in part mediated by CX3CL1 and almost completely abolished when endothelial cells are exposed to higher but still physiological shear stress.

## 4. Discussion

Our present study demonstrates that the exposure of endothelial cells to conditions of high shear stress downregulates CX3CL1 mRNA and protein expression and prevents further upregulation of the chemokine in response to TNF*α*. By this, shear stress reduces the adhesiveness of endothelial cells for monocytes. Vice versa, static conditions or low shear stress allows constitutive expression of CX3CL1 in unstimulated cells and the upregulation of CX3CL1 in response to TNF*α*, which then leads to increased monocytic cell recruitment.

Flow conditions have been shown to regulate a number of inflammatory mediators or adhesion molecules on the transcriptional level in endothelial cells. For example, the chemokine CCL-2/MCP-1 and the adhesion molecule VCAM-1 are downregulated under laminar flow conditions, even when cells are stimulated with TNF*α* [11, 12, 30]. We here show that constitutive and induced expression of the proinflammatory chemokine and adhesion molecule CX3CL1 is downregulated by shear stress. By contrast, we observed that the structurally and functionally related chemokine CXCL16 is not downregulated by flow conditions. This may be explained by the differences in the transcriptional regulation of both mediators. On the one hand, the transcription factor NF-*κ*B seems to be involved in the induction of CX3CL1 by inflammatory stimuli [31]. On the other hand, regulatory activity of the transcription factor AP-1 has only been reported for CXCL16 [32, 33]. It is therefore likely that shear stress can prevent the induction of CX3CL1 by proinflammatory cytokines via an inhibitory mechanism on the involved transcription factors such as NF-*κ*B. A potential mediator would have been KLF2, which is directly induced by shear stress via MEF2 and the MAPK signaling pathway, and has been implicated in the induction of eNOS and in keeping endothelial cells in a noninflammatory state [34–36]. However, we observed no regulation of CX3CL1 in endothelial cells when KLF2 expression was induced with statins or inhibited with siRNA (unpublished observation). It is therefore questionable whether KLF2 negatively regulates CX3CL1, and it appears more likely that other transcriptional regulators suppress CX3CL1 expression under shear stress. Additionally, CX3CL1 can be regulated by TNF*α* on the level of RNA stability [37], and it is possible that this post-transcriptional regulation of CX3CL1 is affected by shear stress.

Monocyte recruitment is mediated by a number of effector molecules including adhesion molecules and chemokines. Of note, laminar shear stress reduces expression of CCL-2 which would result in reduced activation of adhesive mechanisms on monocytic cells such as upregulation of integrins [38, 39]. Furthermore, integrin ligands such as VCAM-1 are downregulated on endothelial cells under flow conditions, which would further decrease cell adhesion [10–12]. In contrast to this mechanism, which requires the activation of monocytic cells, CX3CL1 can induce flow-resistant cell adhesion without the need of prior cell activation [18]. Additionally, CX3CL1 can activate monocytic cells to upregulate integrins and thereby tighten adhesive interactions and also induce transmigration through the endothelial cell layer [19, 40]. By neutralizing surface-expressed CX3CL1 on endothelial cells, we could show that this CX3CL1-mediated mechanism is indeed relevant for monocyte adhesion to endothelial cells. However, we also observed CX3CL1-independent adhesion, which could be more dependent on integrin interaction with VCAM-1 on endothelial cells [41, 42]. Importantly, both CX3CL1- and CX3CL1-independent adhesions were effectively suppressed when endothelial cells were exposed to shear stress. In addition to CX3CL1, CXCL16 can also promote cell adhesion via its receptor. The only known CXCL16 receptor CXCR6 is predominantly expressed on T cells but also on monocytic cells [25, 43]. Yet the chemokine does not undergo regulation by shear stress as seen for CX3CL1. This may reflect that CXCL16 not only has proinflammatory functions but also acts as a scavenger receptor [44] with potentially protective functions in atherosclerotic lesions [24].

In healthy subjects, the expression of CX3CL1 is very low and soluble CX3CL1 is almost absent in the circulation [45]. This may be explained by the present findings showing that physiological flow conditions lower the constitutive expression and release of CX3CL1 by endothelial cells. Thus, to evaluate their physiological function in vitro, it is important to culture endothelial cells under flow conditions. Otherwise, the cells will acquire an inflammatory phenotype, which is more representative for pathophysiological situations.

Several studies have demonstrated that CX3CL1 is upregulated in subjects with vascular diseases [45, 46]. More soluble CX3CL1 can be found in the circulation, and this should at least in part be due to the increased expression and shedding of CX3CL1 by vascular cells. In fact, CX3CL1 is found on endothelial cells within sites of vascular inflammation. Notably, these sites often show disturbed blood flow where the shear stress may become less than 1 dyn/cm^2^. Here, endothelial cells show no proper alignment, more inflammatory phenotype, and more monocyte recruitment. In fact, within the carotid sinus of the brachiocephalic artery, the average shear stress is very low and this is a site of high CX3CL1 expression [27]. Experimental induction of low shear stress in carotid arteries in mice is associated with an upregulation of CX3CL1 expression [28]. Small molecules against CX3CR1 can prevent atherosclerosis in mice [47]. Moreover, knockout or inhibition of CX3CL1 in atherosclerosis-prone mice has demonstrated that the chemokine is critical especially for the recruitment of monocytes at these sites of reduced shear stress [27, 28]. These studies suggest that besides CX3CR1 antagonists, also targeting CX3CL1 on endothelial cells may represent a therapeutic option. The findings of our present study explain this site-specific function of CX3CL1 by providing in vitro evidence that the induction of CX3CL1 mRNA expression is profoundly suppressed by endothelial shear stress and only possible at sites where the shear stress is low or absent. Additionally, our data suggest that CX3CL1 should be implicated in very early states of atherogenesis where the disturbed flow enhances the susceptibility of the still-intact endothelium to respond to inflammatory triggers by the upregulation of CX3CL1. Nevertheless, low shear stress is not sufficient to initiate the inflammatory process since effective CX3CL1 induction requires the presence of inflammatory stimuli. This finding is consistent with the concept that low shear stress is only one of many proinflammatory factors which need to synergize for shifting the balance from atheroprotection towards atherogenesis by upregulating various effector molecules including CX3CL1.

## 5. Conclusion

In summary, our study demonstrates that high laminar shear stress downregulates endothelial CX3CL1 mRNA and protein expression and even attenuates its induction mediated by the inflammatory cytokine TNF*α*. As a result, laminar shear stress reduces the adhesiveness of endothelial cells for CX3CR1-positive monocytes. By this mechanism, shear stress can counteract vascular inflammation.
